# Case report: Subcutaneous *Mycobacterium haemophilum* infection in an immunocompetent patient after lipolysis injections

**DOI:** 10.3389/fmed.2023.1098047

**Published:** 2023-01-23

**Authors:** Linan Ni, Danyang Zou, Hong Yang, Zhiqin Gao, Qian Yu, Lianjuan Yang

**Affiliations:** ^1^Department of Medical Mycology, Shanghai Skin Disease Hospital, Tongji University School of Medicine, Shanghai, China; ^2^Shanghai Skin Disease Hospital, Tongji University School of Medicine, STD Institute, Shanghai, China

**Keywords:** *Mycobacterium haemophilum*, lipolysis injections, medical aesthetics, adverse events, immunocompetent patient, subcutaneous infection

## Abstract

*Mycobacterium haemophilum* is a slow-growing, aerobic mycobacterium that acts as a pathogen in immunocompromised adult patients and immunocompetent children. There are only a few rare cases in the literature describing this species as a cause of subcutaneous infections. Here, we describe a subcutaneous infection caused by *M. haemophilum* in an immunocompetent female after lipolysis injections at an unqualified beauty salon, suggesting that this bacteria can also be a potential causative agent of adverse events in medical aesthetics. In addition, *M. haemophilum* caused lesions not only at the injection sites and adjacent areas but also invaded distant sections through the subcutaneous sinus tracts. Thus, early diagnosis and appropriate treatment are vital to prevent further deterioration and improve prognosis.

## Introduction

*Mycobacterium haemophilum*, a ubiquitous organism, was first isolated in 1978 from subcutaneous lesions of a patient with Hodgkin’s disease (1). It is a slow-growing, aerobic, fastidious mycobacterium that requires a hemoglobin-supplemented medium and a low temperature of 30–32°C for optimal growth (2). Because of these particular culture conditions and the use of inappropriate techniques, it is difficult to isolate and, consequently, few reports can be found in the literature.

In recent years, infections with *M. haemophilum* have been subsequently reported in healthy children with lymphadenitis and immunocompromised patients that had undergone organ transplantation or presented with lymphoma, acquired immunodeficiency syndrome (AIDS), rheumatoid arthritis, marrow hypoplasia, and Crohn’s disease (3). Moreover, *M. haemophilum* can also cause cutaneous infection in healthy adults after trauma, such as tattooing and acupuncture (4).

Herein, we describe a case of subcutaneous infection caused by *M. haemophilum* after lipolysis injections in the upper back and shoulders of an immunocompetent female.

## Report of case

A 34-year-old woman was admitted to our clinic with multiple subcutaneous abscesses and nodules on the back and shoulders. She had a history of multiple subcutaneous lipolysis injections in the upper back and shoulders. The syringes were self-purchased and administered at an unqualified beauty salon. The initial lesions manifested as pinpoint-red spots that subsequently developed into nodules and abscesses that broke down, discharged pus, and crusted. At the time, the patient sought medical care at an unqualified private clinic, where she was diagnosed with a cutaneous infection and empirically treated with a 3-month course of oral doxycycline, roxithromycin, and minocycline. However, the lesions continued to progress and worsen. Unfortunately, the patient had to stop antibiotic therapy during this period because of substantially elevated alanine aminotransferase levels.

**FIGURE 1:**
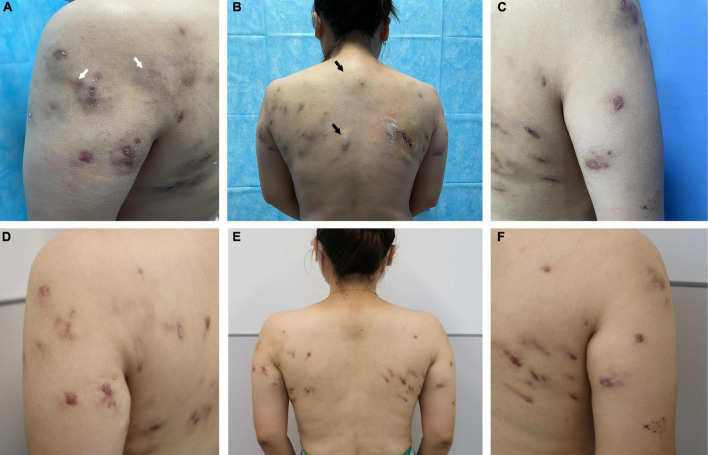
Clinical images. *Mycobacterium haemophilum* infection after lipolysis injection at the initial visit **(A)** in the left shoulder, petechiae, and swelling (white arrows); **(B)** in the back, the area near the spine (not injected) also showed significant occupancy (black arrows); and **(C)** in the right shoulder. **(D–F)** Significant improvement in the patient’s eruption can be observed after treatment.

**FIGURE 2:**
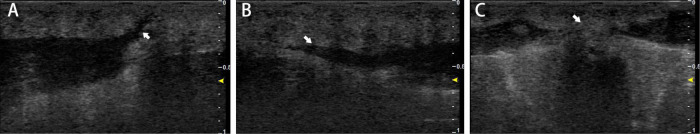
Skin ultrasound image findings. Ultrasonography of the skin lesions revealed multiple mixed cystic and solid areas on the back. Sinus tracts **(A)** opened to the body surface (white arrow), **(B)** spreading outward (white arrow), and **(C)** with a tendency to fuse between two occupancies (white arrow).

The patient was then evaluated at our hospital. She had no significant medical history and was afebrile. Physical examination revealed multiple subcutaneous nodules and abscesses in the shoulders and upper back (Figures 1A, C). While several abscesses ruptured with minor pus exudate, a few continued to invade distant areas and formed new subcutaneous abscesses near the spine, distant from the injection’s initial site (Figure 1B). Superficial regional lymph nodes were not detected. Hematological analysis revealed alanine aminotransferase 39 U/L, triglycerides 2.36 mmol/L, and cholesterol 5.65 mmol/L. Routine blood, urine, and renal function tests showed no abnormalities. Serum markers for human immunodeficiency virus and syphilis were negative. Additionally, the results of cytokine tests, peripheral blood lymphocyte subsets, immune globulin, and complement were within normal limits. The patient’s examination results, electrocardiography, abdominal ultrasound, and lung computerized tomography showed no significant abnormalities. Ultrasonography of the skin lesions revealed multiple mixed cystic and solid areas in the back, along with inflammatory lesions. Internally, the sinus tracts were identical and connected to the body surface (Figure 2A). They even spread outward (Figure 2B), and in some places the pairs tend to fuse between each other (Figure 2C).

**FIGURE 3:**
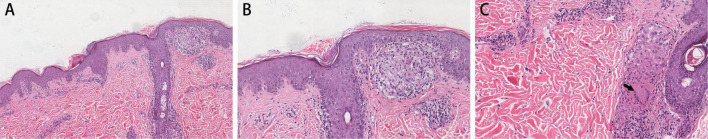
Histopathological findings. Skin lesion Hematoxylin and Eosin staining shows **(A)** epidermal hyperplasia and infectious granuloma into the dermis in certain areas; **(B)** infiltration of epithelioid cells, lymphocytes and other inflammatory cells; and **(C)** multinucleated giant cells (black arrow). Magnification: **(A)** ×200; **(B,C)** ×400.

**FIGURE 4:**
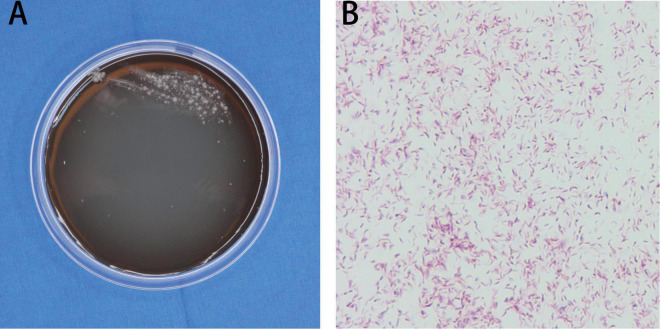
Mycobacterial strain identification. **(A)** Morphology of the skin lesion on Middlebrook 7H11 agar. **(B)** Acid-fast staining showed red and rod-shaped bacteria in branched filaments. Magnification: **(B)** ×1000.

Histopathological examination of the skin tissue specimen showed non-specific, diffuse inflammatory cell infiltration, suggestive of infectious granuloma (Figures 3A–C), but failed to reveal any special strains or organisms using Periodic acid-Schiff and acid-fast staining. The specimens were then inoculated on blood agar, chocolate agar, Lowenstein-Jensen medium, Sabouraud dextrose agar, thioglycollate broth, and Middlebrook 7H11 agar for isolation of pathogenic strains. After 3 weeks of incubation, Middlebrook 7H11 agar exposed offwhite bacterial colonies (Figure 4A). Acid-fast staining revealed red and rod-shaped bacteria and branched filaments (Figure 4B). The isolate was further confirmed as *M. haemophilum* by 16s-rRNA gene sequencing. Finally, the patient was diagnosed with a subcutaneous infection caused by *M. haemophilum*.

The treatment consisted of a combination of antibiotics: oral rifampicin (600 mg/day), clarithromycin (1 g/day), and levofloxacin (500 mg/day) for 6 months, until substantial lesion improvement was seen. Subsequently, *in vitro* antimicrobial susceptibility testing confirmed that the isolate was sensitive to linezolid and amikacin. Considering the higher safety profile of linezolid, the therapy was switched to oral linezolid (1200 mg/day). After a year and a half, the patient’s skin lesions gradually healed, only leaving scars (Figures 1D–F). The patient is still monitored on an ongoing basis.

## Discussion

*Mycobacterium haemophilum* infection occurs mainly in immunocompromised hosts and usually causes skin lesions such as nodules, abscesses, and ulcers (5). This pathogen can invade other organs leading to arthritis, osteomyelitis, conjunctivitis, pneumonia, and disseminated infection, which can be disabling and potentially life-threatening (6). Furthermore, it can also cause skin infections in healthy adults (7). To date, twenty-one cases of cutaneous *M. haemophilum* infections in immunocompetent patients have been reported in the literature (8–14). Among them, fourteen cases were after permanent eyebrow makeup (8, 9), one had a history of trauma (10), one was stabbed by coral (11), one was after tattooing (12), one was after coronary artery bypass grafting (13), and the last three cases, all patients older than 80 years, had some underlying disease and denied history of medical immunosuppression or exogenous trauma (14). In the present case, *M. haemophilum* infection occurred in an immunocompetent patient’s upper body following a cosmetic injection. It is worth noting that *M. haemophilum* infections are prone to occur in the extremities, with lower temperatures to facilitate the pathogen growth (15). Still, our evidence shows that it can also involve the torso, even though it has a higher temperature.

Injectable lipolysis treatment is gradually emerging as a cosmetic procedure to reduce localized fat deposits. The main ingredient of lipolysis injections is phosphatidylcholine (16), injected into the subcutaneous fat layer and with the ability to cause fibrosis and necrosis of adipose tissue (17). In particular, lipolysis procedures are still in clinical development in China with many technical details still under progress, and no pharmaceutical products for injections or treatment have been approved by the State Food and Drug Administration (18). As a result, these injections are commonly used in informal medical aesthetic facilities, where improper procedures and inadequate sterilization usually facilitate infection. Even though *Mycobacterium abscessus* and *Mycobacterium fortuitum* are mycobacterial pathogens often associated with cosmetic injection (19), the causative agent in our case was *M. haemophilum*, suggesting that this species can also cause potential adverse events related to medical aesthetics.

Interestingly, we noted that the lesions in this patient were not only located in the injection site but also invaded distant areas *via* the subcutaneous sinus tract. This phenomenon was confirmed by skin ultrasound imaging. Previous studies indicated that skin lesions related to medical aesthetic injections are mostly confined to injured areas, and disseminated infections are rare in immunocompetent populations contrary to our case (4). We speculate that this might be related to bacterial load, virulence factors, and inappropriate previous treatment. Our patient received empirical antibiotic therapy after a first evaluation that finally failed, reminding us of the necessity of pre-treatment drug sensitivity testing. The selection of sensitive antibiotics is key to improving the prognosis.

Rapid diagnostic of mycobacterial pathogens related to cutaneous infections is challenging and, for this reason, treatment may be delayed resulting in restricted organ function or even disability. Molecular identification of the organism presents a higher advantage for early successful diagnosis. Of course, even after the patients are diagnosed, patients require a long course of anti-mycobacterial treatment (20). Therefore, a high degree of clinical suspicion followed by early successful identification of the organism and selection of proper antibiotics can help diagnose and treat the infection, reducing the morbidity and complications.

## Data availability statement

The original contributions presented in this study are included in the article/supplementary material, further inquiries can be directed to the corresponding authors.

## Ethics statement

Written informed consent was obtained from the individual(s) for the publication of any potentially identifiable images or data included in this article.

## Author contributions

LN: manuscript design and draft, data analysis, acquisition, and interpretation. HY, ZG, and DZ: laboratory testing and data analysis. QY and LY: study design, revision, and manuscript finalization. All authors contributed to the manuscript and approved the submitted version.

## References

[1] Sompolinsky D Lagziel A Naveh D Yankilevitz T. *Mycobacterium haemophilum* sp. nov., a new pathogen of humans. *Int J Syst Bacteriol.* (1978) 28:67–75. 10.1099/00207713-28-1-67

[2] Dawson D Jennis F. Mycobacteria with a growth requirement for ferric ammonium citrate, identified as *Mycobacterium haemophilum*. *J Clin Microbiol.* (1980) 11:190–2. 10.1128/jcm.11.2.190-192.1980 7358843 PMC273352

[3] Saubolle M Kiehn T White M Rudinsky M Armstrong D. *Mycobacterium haemophilum*: microbiology and expanding clinical and geographic spectra of disease in humans. *Clin Microbiol Rev.* (1996) 9:435–47. 10.1128/CMR.9.4.435 8894345 PMC172903

[4] Weitzul S Eichhorn P Pandya A. Nontuberculous mycobacterial infections of the skin. *Dermatol Clin.* (2000) 18:359–77. 10.1016/s0733-8635(05)70182-0 10791163

[5] Greenwood J Nielsen N Miller N. Patient on immunomodulatory therapy experiencing joint pain and skin lesions: a case report. *J Prim Care Commun Health.* (2021) 12:21501327211005894. 10.1177/21501327211005894 33764183 PMC8772354

[6] Fairhurst R Kubak B Pegues D Moriguchi J Han K Haley J *Mycobacterium haemophilum* infections in heart transplant recipients: case report and review of the literature. *Am J Trans.* (2002) 2:476–9. 10.1034/j.1600-6143.2002.20514.x 12123216

[7] Groves R. Unusual cutaneous mycobacterial diseases. *Clin Dermatol.* (1995) 13:257–63. 10.1016/0738-081x(95)00003-x 8521367

[8] Giulieri S Morisod B Edney T Odman M Genné D Malinverni R Outbreak of *Mycobacterium haemophilum* infections after permanent makeup of the eyebrows. *Clin Infect Dis.* (2011) 52:488–91. 10.1093/cid/ciq191 21258102

[9] Wollina U. Nodular skin reactions in eyebrow permanent makeup: two case reports and an infection by *Mycobacterium haemophilum*. *J Cosmet Dermatol.* (2011) 10:235–9. 10.1111/j.1473-2165.2011.00564.x 21896137

[10] Bao F Yu C Pan Q Liu Y Zhou G Liu H Cutaneous *Mycobacterium haemophilum* infection in an immunocompetent patient. *J Deutschen Dermatol Gesellschaft.* (2020) 18:1186–8. 10.1111/ddg.14177 32666629

[11] Smith S Taylor G Fanning E. Chronic cutaneous *Mycobacterium haemophilum* infection acquired from coral injury. *Clin Infect Dis.* (2003) 37:e100–1. 10.1086/377267 13130418

[12] Kay M Perti T Duchin J. Tattoo-associated *Mycobacterium haemophilum* skin infection in immunocompetent adult, 2009. *Emerg Infect Dis.* (2011) 17:1734–6. 10.3201/eid1709.102011 21888807 PMC3322073

[13] McBride M Rudolph A Tschen J Cernoch P Davis J Brown B Diagnostic and therapeutic considerations for cutaneous *Mycobacterium haemophilum* infections. *Arch Dermatol.* (1991) 127:276–7. 10.1001/archderm.1991.01680020148035 1991007

[14] Tan W Tan K Tay Y Ang C. Erosive and granulomatous forearm dermatitis of the elderly, a new presentation for cutaneous *Mycobacterium haemophilum* infection: a brief report. *Int J Dermatol.* (2021) 60:e309–11. 10.1111/ijd.15484 33650117

[15] Lindeboom J Bruijnesteijn van Coppenraet LE van Soolingen D Prins JM Kuijper EJ. Clinical manifestations, diagnosis, and treatment of *Mycobacterium haemophilum* infections. *Clin Microbiol Rev.* (2011) 24:701–17. 10.1128/CMR.00020-11 21976605 PMC3194825

[16] Duncan D Palmer M. Fat reduction using phosphatidylcholine/sodium deoxycholate injections: standard of practice. *Aesthetic Plast Surg.* (2008) 32:858–72. 10.1007/s00266-008-9188-9 18612680

[17] Park E Kim H Kim M Oh H. Histological changes after treatment for localized fat deposits with phosphatidylcholine and sodium deoxycholate. *J Cosmet Dermatol.* (2013) 12:240–3. 10.1111/jocd.12053 23992167

[18] Chen S Wang J Chen B Wang F Zhang R Gao Q, et al. Treatment strategy of non-tuberculous *Mycobacterium* infection caused by facial lipolytic injection. *Chin J Aesthet Med.* (2017) 26:4.

[19] Lim J Kim J Yang H. Management of infections with rapidly growing mycobacteria after unexpected complications of skin and subcutaneous surgical procedures. *Arch Plast Surg.* (2012) 39:18–24. 10.5999/aps.2012.39.1.18 22783486 PMC3385308

[20] Chung J Ince D Ford B Wanat K. Cutaneous infections due to nontuberculosis *Mycobacterium*: recognition and management. *Am J Clin Dermatol.* (2018) 19:867–78. 10.1007/s40257-018-0382-5 30168084

